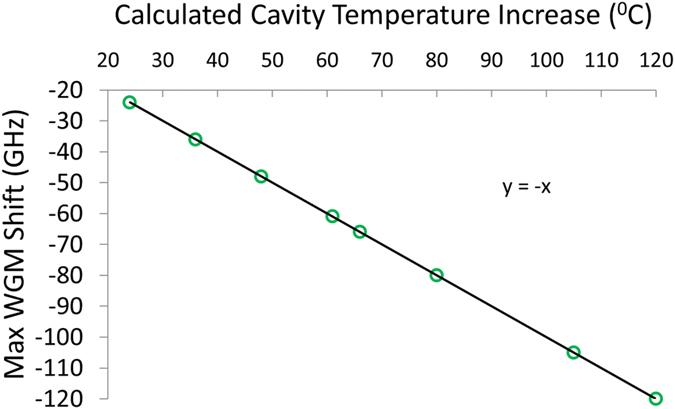# Corrigendum: Glass-on-Glass Fabrication of Bottle-Shaped Tunable Microlasers and their Applications

**DOI:** 10.1038/srep39437

**Published:** 2017-01-23

**Authors:** Jonathan M. Ward, Yong Yang, Síle Nic Chormaic

Scientific Reports 6: Article number: 2515210.1038/srep25152; published online: 04 28 2016; updated: 01 23 2017

This Article contains an error in Figure 4b, which uses an inaccurate value for the glass thermo-optic coefficient.

The thermo-optic coefficient stated in the Article is dn/dT = −21*10^−7^K^−1^, however the value stated by the manufacturer is dn/dT = −63*10^−7^K^−1^ (for 20–40 °C). The calculated temperature of the resonator for a thermo-optic coefficient of −63*10^−7^K^−1^ (20–40 °C) changes the calculated mode shift rate from ~1.9 GHz/K to ~1 GHz/K, implying the cavity temperature is almost doubled.

The data points in Figure 4c are not affected, however the fitting curves are. These can be readjusted by modifying the fitting parameters to take into account the increased temperature. This can be done by altering the thermal expansion co-efficient from 114*10^−7^K^−1^ (20–40 °C) to 119*10^−7^K^−1^. The fitting parameter, n, remains as 0.84 by slightly decreasing the empirical calibration constants (A and B). The resulting change to the fitting curves is negligible.

The thermo-optic coefficient and the thermal expansion coefficient quoted by the manufacturer are given for a specific temperature range only (for 20–40 °C) and are known to vary outside this range. The authors’ glass resonator is operating well outside this temperature range and so it is reasonable to adjust these coefficients for the purpose of fitting.

The correct Figure 4b appears below as Figure 1.

## Figures and Tables

**Figure 1:**